# Assessment of fluid responsiveness in spontaneously breathing patients: a systematic review of literature

**DOI:** 10.1186/s13613-018-0365-y

**Published:** 2018-02-09

**Authors:** Renato Carneiro de Freitas Chaves, Thiago Domingos Corrêa, Ary Serpa Neto, Bruno de Arruda Bravim, Ricardo Luiz Cordioli, Fabio Tanzillo Moreira, Karina Tavares Timenetsky, Murillo Santucci Cesar de Assunção

**Affiliations:** 1 Intensive Care Unit, Hospital Israelita Albert Einstein, Av. Albert Einstein, 627/701, 5th Floor, São Paulo, SP 05651-901 Brazil; 2 Intensive Care Unit, Hospital Municipal Dr. Moysés Deutsch - M’Boi Mirim, São Paulo, SP Brazil; 3 Department of Intensive Care, Academic Medical Center, University of Amsterdam, Amsterdam, The Netherlands

**Keywords:** Fluid responsiveness, Spontaneously breathing, Echocardiography, Stroke volume, Pulse pressure, Intensive care, Critical care

## Abstract

Patients who increase stoke volume or cardiac index more than 10 or 15% after a fluid challenge are usually considered fluid responders. Assessment of fluid responsiveness prior to volume expansion is critical to avoid fluid overload, which has been associated with poor outcomes. Maneuvers to assess fluid responsiveness are well established in mechanically ventilated patients; however, few studies evaluated maneuvers to predict fluid responsiveness in spontaneously breathing patients. Our objective was to perform a systematic review of literature addressing the available methods to assess fluid responsiveness in spontaneously breathing patients. Studies were identified through electronic literature search of PubMed from 01/08/2009 to 01/08/2016 by two independent authors. No restrictions on language were adopted. Quality of included studies was evaluated with Quality Assessment of Diagnostic Accuracy Studies tool. Our search strategy identified 537 studies, and 9 studies were added through manual search. Of those, 15 studies (12 intensive care unit patients; 1 emergency department patients; 1 intensive care unit and emergency department patients; 1 operating room) were included in this analysis. In total, 649 spontaneously breathing patients were assessed for fluid responsiveness. Of those, 340 (52%) were deemed fluid responsive. Pulse pressure variation during the Valsalva maneuver (∆PPV) of 52% (AUC ± SD: 0.98 ± 0.03) and passive leg raising-induced change in stroke volume (∆SV-PLR) > 13% (AUC ± SD: 0.96 ± 0.03) showed the highest accuracy to predict fluid responsiveness in spontaneously breathing patients. Our systematic review indicates that regardless of the limitations of each maneuver, fluid responsiveness can be assessed in spontaneously breathing patients. Further well-designed studies, with adequate simple size and power, are necessary to confirm the real accuracy of the different methods used to assess fluid responsiveness in this population of patients.

**Electronic supplementary material:**

The online version of this article (10.1186/s13613-018-0365-y) contains supplementary material, which is available to authorized users.

## Background

Intravascular volume expansion is a common intervention in critically ill patients [1]. Patients who will benefit from intravascular volume expansion, i.e., will boost stroke volume (SV) after a volume expansion, have both ventricles in the ascending portion of the Frank–Starling curve, characterizing a preload dependency [1, 2]. Nevertheless, nearly 50% of critically ill patients will not benefit from an intravascular volume expansion [2, 3]. Conversely, an accurate assessment of fluid responsiveness prior to volume expansion is critical to avoid fluid overload, which has been associated with increased morbidity and mortality in critically ill patients [4–6].

The concept of predicting fluid responsiveness was initially reported in deeply sedated patients under volume-controlled mechanical ventilation with tidal volume (VT) of at least 8 ml/Kg and positive end-expiratory pressure (PEEP) lower than 10 cm H_2_O [7]. Nonetheless, since many patients in the intensive care unit (ICU) are not under such conditions, for many years the presence of spontaneous breathing or inspiratory efforts, with or without an endotracheal tube, was considered a major limitation to assess fluid responsiveness in critically ill patients [8].

Knowledge on the interaction between heart, lung and abdominal compartment is critical to understanding the concept of fluid responsiveness [9, 10]. In spontaneous breathing patients without mechanical ventilation, intrathoracic pressure decreases, while venous return and stroke volume increases during inspiration [10]. On the other hand, at expiration, intrathoracic pressure increases, while venous return and stroke volume decreases [10]. Thus, quantifying stroke volume variation, between respiratory cycles could be used to assess fluid responsiveness [1].

Static [11, 12] and dynamic [8, 13] parameters have been proposed to assess fluid responsiveness in critically ill patients. The available evidence clearly shows that dynamic parameters exhibited a higher accuracy than static parameters to predict fluid responsiveness [13, 14]. Pulse pressure variation, [15–20] echocardiography maneuvers [21–28] and passive leg raising [18, 21–23, 25, 27, 29] are tools that could be used to assess fluid responsiveness in spontaneously breathing patients.

Thus, our primary objective was to perform a systematic review addressing the available methods for fluid responsiveness assessment in spontaneously breathing patients. A secondary objective was to summarize the performance of available methods to assess fluid responsiveness in spontaneously breathing patients.

## Methods

This systematic review was reported following the PRISMA (Preferred Reporting Items for Systematic Reviews and Meta-Analyses) guidelines [30].

### Eligibility criteria

Articles were selected for inclusion if they evaluated fluid responsiveness in spontaneous breathing adult patients. Articles were assessed for eligibility if one of the following standard definitions of fluid responsiveness and fluid challenge was adopted: increase in stroke volume (SV) ≥ 10% and/or cardiac output (CO) ≥ 10% and/or cardiac index (CI) [31] ≥ 10% and/or aortic velocity–time integral (VTI) ≥ 10% after a fluid challenge [2, 32]. Fluid challenge was considered adequate if at least 250 ml over 30 min of intravenous (I.V.) fluid was infused [2, 33]. Spontaneously breathing was defined as patients without any ventilatory support, patients on noninvasive mechanical ventilation or patients on invasive mechanical ventilation in a spontaneous mode. Patients in the following clinical scenarios were included: ICU, emergency department (ED) and operating room.

### Identifying studies

An electronic literature search was carried out by two authors through a computerized blinded search on PubMed. The following search strategy was applied: (((“hemodynamics”[MeSH Terms] OR “hemodynamics”[All Fields]) AND (“respiration”[MeSH Terms] OR “respiration”[All Fields] OR “cell respiration”[MeSH Terms] OR (“cell”[All Fields] AND “respiration”[All Fields]) OR “cell respiration”[All Fields]) AND (“cardiac output”[MeSH Terms] OR (“cardiac”[All Fields] AND “output”[All Fields]) OR “cardiac output”[All Fields]))). Literature search was limited to a period of time (01/08/2009 to 01/08/2016) and to “human.” No restrictions on language were adopted. Additionally, we hand-searched the reference lists of the included studies to identify other relevant studies.

### Study selection

Prospective studies that reported sensitivity, specificity, cutoff value of each maneuver to assess fluid responsiveness, number of patients included and frequency of fluid responsiveness and non-fluid responsiveness patients were included in this systematic review. Review articles, editorials, studies assessing fluid responsiveness during mechanical ventilation and studies that did not report outcomes of interest were excluded.

### Data extraction

Two authors independently screened all retrieved citations by reviewing their titles and abstracts (RCFC and FTM). Then, the reviewers independently evaluated the full-text manuscripts for eligibility using a standardized form. Reviewers independently extracted the relevant data from the full-text manuscripts and assessed the risk of bias using a standardized form. Any disagreement between the authors was resolved by a third author (ASN).

### Quality assessment

The quality of each study was evaluated by the Quality Assessment of Diagnostic Accuracy Studies tool (QUADAS) [34]. Details of the quality assessment are reported in Additional file 1.

### Primary objective

The primary objective was to report the available methods to assess fluid responsiveness in spontaneously breathing patients.

### Secondary objectives

Secondary objectives were to assess diagnostic performance and build a receiver operating characteristics curve (ROC curve) of methods available to assess fluid responsiveness in spontaneously breathing patients.

### Methods for fluid responsiveness assessment

Assessed methods to predict fluid responsiveness were pulse pressure variation (∆PP); [15, 17, 19] systolic pressure variation (∆SP); [15] ∆PP during forced inspiratory effort (∆PPf); [15] ∆SP during forced inspiratory effort (∆SPf); [15] ∆PP during the Valsalva maneuver (∆PPV); [16] ∆SP during the Valsalva maneuver (∆VSP); [16] lowest pulse pressure (PPmin); [16] stroke volume variation (∆SV); [17, 21, 26] passive leg raising (PLR)-induced change in stroke volume (∆SV-PLR); [18, 23, 29] PLR-induced change in radial pulse pressure (∆PP-PLR); [18] PLR-induced change in the velocity peak of femoral artery flow (∆VF-PLR); [18] deep inspiration maneuver-induced change in pulse pressure (∆PPdim); [19] respiratory change in velocity peak of femoral artery flow (∆VF); [19] deep inspiration maneuver-induced change in velocity peak of femoral artery flow (∆VFdim); [19] ∆PP during forced inspiratory breathing (∆PP_FB_); [20] PLR-induced change in stroke volume index (SVi-PLR); [21] change in cardiac output (ΔCO); [22] inferior vena cava collapsibility index (cIVC); [24, 26–28] E wave velocity; [24] aortic velocity time index (VTI) variations during PLR (∆VTI-PLR); [25] VTI ≤ 21 cm; [25] aortic velocity variation (AoVV); [26] inferior vena cava maximum diameter (IVCmax); [27] ∆CO between baseline and after PLR (∆CO-PLR) [27].

Pulse pressure variation was calculated as the difference in pulse pressure maximal (PPmax) and pulse pressure minimal (PPmin) over the respiratory cycle divided by the mean between PPmax and PPmin [∆PP = (PPmax − PPmin)/(PPmax + PPmin)/2] [16, 19, 20]. Passive leg raising consists in moving the patient from the 45° semirecumbent position to a horizontal position with the lower limbs lifted 30°–45° relative to the trunk [1, 18]. PLR was determined as the difference between baseline and the highest value induced during the PLR or after the PLR [21, 23, 27]. Inferior vena cava collapsibility index represents the difference in the vena cava maximum diameter (IVCmax) and vena cava minimum diameter (IVCmin) divided by the vena cava maximum diameter over the respiratory cycle [cIVC = (IVCmax − IVCmin)/(IVCmax)] [26, 27]. Valsalva maneuver consists of sustaining a forced expiration effort against a closed mouth [16]. Forced inspiratory breaths consist of three respiratory cycles of deep inspiration immediately followed by slow passive expiration [20]. Deep inspiration maneuver consists of slow continuous inspiration strain (5–8 s) followed by slow passive exhalation [19].

### Statistical analysis

The number of patients included, study design, setting, inclusion and exclusion criteria, time and type of fluid infused, the best cutoff value of each maneuver and definition of fluid responders were extracted from published studies. The accuracy of each diagnostic test was assessed with sensitivity (Sens), specificity (Spec), positive predictive value (PPV), negative predictive value (NPV), positive likelihood ratio (LR +), negative likelihood ratio (LR −), AUC along with its standard deviation (SD) or 95% confidence interval (95% CI). Whenever not reported, accuracy, PPV, NPV, LR + and LR − were calculated using the Review Manager (RevMan) [computer program]—version 5.3—Copenhagen: The Nordic Cochrane Centre, The Cochrane Collaboration, 2014.

A receiver operator characteristics curve (ROC curve) was constructed using the sensitivity and specificity of each maneuver extracted from included study using Meta-DiSc version 1.4 (Universidad Complutense, Madrid, Spain) [35]. Methods for fluid responsiveness assessment were classified according to their accuracy [area under the receiver operating characteristics curve (AUC)]. AUC from 0.90 to 1.00 was considered excellent, from 0.80 to 0.89 adequate, from 0.70 to 0.79 fair, from 0.60 to 0.69 poor and from 0.50 to 0.59 failure [36].

## Results

### Search results

**Fig. 1:**
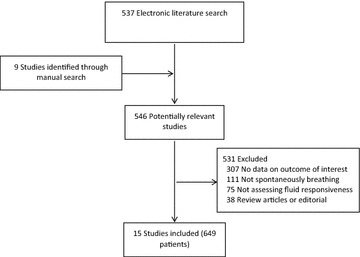
Literature search strategy

The initial search strategy identified 537 studies (Fig. 1). After screening the reference lists of the included studies, 9 potentially relevant articles were included and 546 potentially relevant articles were selected. Fifteen prospective studies (649 patients in total) were included in this systematic review after the exclusion of 531 studies (307 studies had no data on outcome of interest, 111 studies did not regard spontaneously breathing patients, 75 studies did not access fluid responsiveness, and 38 were review articles or editorials) (Fig. 1).

### Characteristics of included studies

Characteristics of included studies are presented in Tables 1 [15–20] and 2 [21–29]. Out of fifteen studies included, twelve evaluated fluid responsiveness in ICU patients, [15–19, 21–27] one included ED patients, [29] one included ICU and ED patients [28] and one included operating room patients (elective thoracic surgery) [20] (Tables 1 and 2).

**Table 1:** Characteristics of included studies addressing pulse pressure variation for fluid responsiveness in spontaneously breathing patients

Author, year	*N*	Setting	Inclusion criteria	Exclusion criteria	Ventilation	Fluid challenge	Definition of responders	Maneuvers
Soubrier, 2007 [15]	32	ICU	1. Low blood pressure 2. Tachycardia 3. Oliguria 4. Mottled skin	1. Arrhythmia 2. Lack of cooperation	SB	500 ml I.V. 6% HES over 20 min	↑CI ≥ 15%	1. ∆PP 2. ∆SP 3. ∆PPf 4. ∆SPf
M. García, 2009 [16]	30	ICU	1. Hypotension 2. Tachycardia 3. Oliguria	1. Arrhythmia 2. History of syncope 3. Lack of cooperation	SB	500 ml I.V. 6% HES over 30 min	↑SVi > 15%	1. ∆PPV by PCA 2. ∆VSP by PCA 3. PPmin
Monnet, 2009 [17]	23	ICU	1. SBP < 90 mmHg 2. Tachycardia 3. UO < 0.5 ml/kg/h 4. Mottled skin	1. Not sustain an inspiration for over 15 seconds	SB and SBmv	500 ml I.V. saline over 10 min	↑CI > 15%	1. ∆PP by PCA 2. ∆SV by PCA
Préau, 2010 [18]	34	ICU	1. SBP < 90 mmHg 2. Tachycardia 3. UO < 0.5 mL/kg/h 4. Mottled skin	1. Arrhythmia 2. Aortic insufficiency 3. VNI was warranted	SB	500 mL I.V. 6% HES over 30 min	↑SV ≥ 15%	1. ∆SV-PLR by TE 2. ∆PP-PLR 3. ∆VF-PLR by Doppler
Préau, 2012 [19]	23	ICU	1. SBP < 90 mmHg 2. Tachycardia 3. Regular cardiac rhythm 4. UO < 0.5 mL/kg/h	1. RR > 30 2 Not sustain an inspiration for over 5 s 3. Aortic insufficiency 4. MV was warranted	SB	500 mL I.V. 6% HES over 30 min	↑SV > 15%	1. ∆PP 2 ∆PPdim 3. ∆VF by Doppler 4. ∆VFdim by Doppler
Hong, 2014 [20]	59	OP	1. Age 18–80 years 2. Elective thoracic surgery	1. Arrhythmia 2. Intracardiac shunt 3. Valvulopathy 4 Cardiac or pulmonary dysfunction	SB	6 ml/kg of I.V. HES for 10 min	↑CI ≥ 15%	1. ∆PP_FB_ by PCA

*ICU* intensive care unit, *OP* operating room, *SBP* systolic blood pressure, *UO* urine output, *VNI* ventilation noninvasive, *RR* respiratory rate, *MV* mechanical ventilation, *COPD* chronic obstructive pulmonary disease, *SB* spontaneous breathing without any ventilatory support, *SBmv* mechanical ventilation during spontaneous mode, *I.V*. intravenous, *HES* hydroxyethyl starch, ↑ = increase, *CI* cardiac index, *SV* stroke volume, *∆PP* pulse pressure variation, *∆SP* systolic pressure variation, *∆PPf* ∆PP during forced inspiratory effort, *∆SPf* ∆SP during forced inspiratory effort, *∆PPV* ∆PP during the Valsalva maneuver, *PCA* pulse contour analysis, *∆VSP* ∆SP during the Valsalva maneuver, *PPmin* lowest pulse pressure, *∆SV* stroke volume variation, *PLR* passive leg raising, *∆SV-PLR* PLR-induced change in stroke volume, *∆PP-PLR* PLR-induced change in radial pulse pressure, *∆VF-PLR* PLR-induced change in the velocity peak of femoral artery flow, *∆PPdim* deep inspiration maneuver-induced change in pulse pressure, *∆VF* respiratory change in velocity peak of femoral artery flow, *∆VFdim* deep inspiration maneuver-induced change in velocity peak of femoral artery flow, *∆PP*_*FB*_ ∆PP during forced inspiratory breathing

**Table 2:** Characteristics of included studies addressing echocardiography maneuvers, pulse contour analysis or noninvasive cardiac output monitor (NICOM^®^) for fluid responsiveness in spontaneously breathing patients

Author, year	*N*	Setting	Inclusion criteria	Exclusion criteria	Ventilation	Fluid challenge	Definition of responders	Maneuvers
Lamia, 2007 [21]	24	ICU	1. MAP < 60 mmHg 2. Tachycardia 3. UO < 0.5 ml/kg/h 4. Delayed CRT	1. Aortic valvulopathy 2. Mitral insufficiency or stenosis	SB and SBmv	500 ml I.V. saline for 15 min	↑SVi ≥ 15%	1. SVi-PLR by TE
Maizel, 2007 [22]	34	ICU	1. Hypotension 2. Acute renal failure 3. Dehydration	1. Hemorrhage 2. PLR contraindications 3. Arrhythmia	SB	500 ml I.V. saline over 15 min	↑CO ≥ 12%	1. ∆CO-PLR by TE 2. ∆SV-PLR by TE
Biais, 2009 [23]	30	ICU	1. SBP < 90 mmHg 2. Tachycardia 3. Acute renal failure 4 Mottled skin	1. ↑ intra-abdominal pressure 2. BMI < 15 or > 40 kg/m2 3. Valvulopathy 4 Intracardiac shunt	SB and SBmv	500 ml I.V. saline for 15 min	↑SV > 15%	1. ∆SV-PLR_TE_ by TE 2. ∆SV-PLR_FloT_ by PCA
Muller, 2012 [24]	40	ICU	1. MAP < 65 mmHg 2. Tachycardia 3. UO < 0.5 mL/Kg/h 4. Mottled skin	1. Pulmonary edema 2. Right ventricular failure 3. Elevated left atrial pressure	SB	500 mL I.V. 6% HES over 15 min	↑VTI ≥ 15%	1. cIVC by TE 2. E wave velocity by TE
Brun, 2013 [25]	23	ICU	1. Severe preeclampsia	1. Cardiac or renal disorders prior to pregnancy	SB	500 ml I.V. saline over 15 min	↑SVi ≥ 15%	1. ∆VTI-PLR 2. VTI
Lanspa, 2013 [26]	14	ICU	1. Age ≥ 14 years 2. Infection and SIRS 3. Refractory hypotension	1. Pregnancy 2. Aortic stenosis 3. Arrhythmia 4. COPD and asthma	SB	10 mL/kg of I.V. crystalloids over 20 min	↑CI ≥ 15%	1. cIVC by TE 2. ∆SV by PCA 3. AoVV by TE
Airapetian, 2015 [27]	59	ICU	1. Physician decided to perform fluid expansion	1. Hemorrhage 2. Arrhythmia 3. Compression stockings 4. PLR contraindications	SB	PLR and 500 ml I.V. saline over 15 min	↑CO ≥ 10%	1. cIVC by TE 2. IVCmax by TE 3. ΔCO-PLR by TE
Duus, 2015 [29]	100	ED	1. Age ≥ 18 years 2. Clinical team intended to administer IV fluid	1. Acuity precluding participation in research 2 PLR contraindications	SB	5 ml/kg I.V. saline	↑SV > 10%	1. ∆SV-PLR using NICOM^®^
Corl, 2017 [28]	124	ED and ICU	1. PAS < 90 mmHg 2. Tachycardia 3. UO < 0.5 ml/kg/h 4. Hypoperfusion	1. Cardiogenic, obstructive or neurogenic shock 2. Age < 18 years 3. Hospitalization for > 36 h	SB	500 ml I.V. saline	↑CI ≥ 10%	1. cIVC by TE

*ICU* intensive care unit, *ED* emergency department, *MAP* mean arterial pressure, *UO* urine output, *CRT* capillary refill time, *SBP* systolic blood pressure, *PLR* passive leg raising, ↑ = increase, *BMI* body mass index, *COPD* chronic obstructive pulmonary disease, *SB* spontaneous breathing without any ventilatory support, *SBmv* mechanical ventilation during spontaneous mode, *I.V.* intravenous, *HES* hydroxyethyl starch, *SV* stroke volume, *CO* cardiac output, *VTI* aortic velocity–time integral, *SVi* stroke volume index, *CI* cardiac index, *PLR* passive leg raising, *SVi-PLR* PLR-induced change in stroke volume index, *TE* transthoracic echocardiography, *ΔCO* change in cardiac output, *ΔCO-PLR* ΔCO between baseline and after PLR, *∆SV* stroke volume variation, *∆SV-PLR* PLR-induced change in stroke volume, *FloT* FloTrac™, *PCA* pulse contour analysis, *cIVC* inferior vena cava collapsibility index, *VTI* aortic velocity–time integral, *∆VTI-PLR* VTI variations during PLR, *AoVV* aortic velocity variation, *NICOM*^®^ noninvasive cardiac output monitor, *IVCmax* inferior vena cava maximum diameter

Out of 649 spontaneously breathing patients assessed for fluid responsiveness, 340 patients (52%) were responders. In 12 studies [12/15 (80%)], only spontaneous breathing patients without any type of ventilatory support were included (572 patients) [15, 16, 18–20, 22, 24–29]. Out of those, 51% (291/572) of patients without ventilatory support were considered fluid responsive (Tables 1 and 2). In 3 studies [3/15 (20%)], spontaneous breathing patient without any ventilatory support and patients under mechanical ventilation in a spontaneous mode were included (77 patients) [17, 21, 23]. Of those, 63% (49/77) patients were deemed responsive to a fluid challenge (Tables 1 and 2).

### Fluid challenge characteristics

Fluid challenge was performed in seven (46.6%) studies through an I.V. infusion of 500 ml of saline; [17, 21–23, 25, 27, 28] five studies (33.3%) with 500 ml of hydroxyethyl starch (HES); [15, 16, 18, 19, 24] one (6.7%) study with 6 ml/kg of HES; [20] one (6.7%) study applied 10 mL/kg of crystalloid; [26] and one (6.7%) study used 5 ml/kg saline [29] (Tables 1 and 2).

Adopted definitions of fluid responsiveness were an increase in SV > 10% [29] or > 15%; [18, 19, 23] an increase in stroke volume index (SVi) ≥ 15%; [16, 21, 25] an increase in CI ≥ 10% [28] or ≥ 15%; [15, 17, 20, 26] an increase in CO ≥ 10% [27] or 12% [22] or an VTI ≥ 15% [24] (Tables 1 and 2). The triggers for intravascular volume expansion varied across the studies and are presented in Tables 1 and 2.

### Methods for fluid responsiveness assessment

Thirty-four maneuvers for predicting fluid responsiveness in spontaneously breathing patients were reported (Tables 1 and 2). Studies that adopted pulse pressure variation to assess fluid responsiveness are summarized in Table 1. Studies that adopted echocardiography maneuvers, pulse contour analysis or noninvasive cardiac output monitor (NICOM^®^) are summarized in Table 2.

### Performance of maneuvers for predicting fluid responsiveness

#### Pooled analysis (15 studies; 649 patients)

**Fig. 2:**
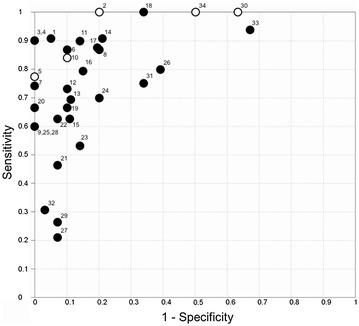
Receiver operating characteristics curve with all methods found in the literature search of assessment volume responsiveness in spontaneous breathing patients. Closed circles represent studies including spontaneous breathing patients without ventilator support; open circles represent studies including patients under mechanical ventilation during spontaneous mode and spontaneous breathing without ventilator support. 1 = ∆PPV of 52%; 2 = ∆SV-PLR_TTE_ >13%; 3 = ∆PPdim ≥12%; 4 = ∆VFdim ≥12%; 5 = SVi-PLR ≥12.5%; 6 = ∆SV-PLR ≥10%; 7 = ∆VTI-PLR >12%; 8 = ∆VF-PLR ≥8%; 9 = ∆SV ≥17%; 10 = ∆SV-PLR_FloT_ >16%; 11 = ∆PP_FB_ = 13.7%; 12 = ∆VSP of 30%; 13 = ∆SV >12%; 14 = PPmin of 45mmHg; 15 = ∆CO >12%; 16 = ∆PP-PLR ≥9%; 17 = cIVC of 25%; 18 = cIVC ≥15%; 19 = E wave velocity of 0.7; 20 = VTI ≤21cm; 21 = ∆SP of 9%; 22 = ∆PP of 12%; 23 = ΔCO-PLR >10%; 24 = cIVC =40%; 25 = ∆VF ≥10%; 26 = ∆SV-PLR; 27 = ∆PPf of 33%; 28 = ∆PP ≥10%; 29 = ∆SPf of 30%; 30 = ∆PP ≥11%; 31 = AoVV ≥25%; 32 = cIVC >42%, 33 = IVCmax <2.1cm, 34 = ∆SV≥10%

Out of 34 reported maneuvers for predicting fluid responsiveness in spontaneously breathing patients, 13 (38%) maneuvers had excellent accuracy (AUC from 0.9 to 1), 9 (26%) had adequate accuracy (AUC from 0.8 to 0.89), 6 (18%) had fair accuracy (AUC from 0.7 to 0.79), 5 (15%) had poor accuracy (AUC from 0.6 to 0.69) and 1 maneuver (3%) was classified as failure (AUC from 0.5 to 0.59) (Fig. 2) (Tables 3 and 4).

**Table 3:** Performance of included studies that addressed pulse pressure variation to predict fluid responsiveness in spontaneously breathing patients

Author, Year	Maneuver	Sens (%)	Spec (%)	PPV (%)	NPV (%)	LR +	LR−	AUC ± SD or (95% CI)
Soubrier, 2007 [15]	∆PP of 12%	63	92	92	63	8.20	0.39	0.81. ± 0.08
	∆SP of 9%	47	92	90	54	6.15	0.57	0.82 ± 0.08
	∆PPf of 33%	21	92	80	44	3.01	0.85	0.72 ± 0.09
	∆SPf of 30%	26	92	83	46	3.75	0.80	0.69 ± 0.10
M. García, 2009 [16]	∆PPV of 52%	91	95	91	95	17,3	0.01	0.98 ± 0.03
	∆VSP of 30%	73	90	80	85	6.91	0.30	0.90 ± 0.07
	PPmin of 45 mmHg	91	79	71	94	4.32	0.12	0.89 ± 0.06
Monnet, 2009 [17]	∆PP ≥ 11%	100	37	80	100	1.75		0.68 (0.45–0.88)
	∆SV ≥ 10%	100	50	84	100	2.00		0.57 (0.34–0.78)
Préau, 2010 [18]	∆SV-PLR ≥ 10%	86	90	86	90	8.57	0.16	0.94 ± 0.04
	∆PP-PLR ≥ 9%	79	85	79	85	5.24	0.25	0.86 ± 0.08
	∆VF-PLR ≥ 8%	86	80	75	89	4.29	0.18	0.93 ± 0.04
Préau, 2012 [19]	∆PP ≥ 10%	60	100	100	76		0.40	0.71. ± 0.12
	∆PPdim ≥ 12%	90	100	100	93		0.10	0.95 ± 0.05
	∆VF ≥ 10%	60	100	100	76		0.40	0.74 ± 0.11
	∆VFdim ≥ 12%	90	100	100	93		0.10	0.95 ± 0.05
Hong, 2014 [20]	∆PP_FB_ = 13.7%	90	87	87	90	6.72	0.12	0.91 (0.80–0.96)

*Sens* sensitivity, *Spec* specificity, *PPV* positive predictive value, *NPV* negative predictive value, *LR* + positive likelihood ratio, *LR* − negative likelihood ratio, *AUC* area under the receiver operating characteristics curve, *SD* standard deviation, *95% CI* 95% confidence intervals, *∆PP* pulse pressure variation, *∆SP* systolic pressure variation, *∆PPf* ∆PP during forced inspiratory effort, *∆SPf* ∆SP during forced inspiratory effort, *∆PPV* ∆PP during the Valsalva maneuver, *∆VSP* ∆SP during the Valsalva maneuver, *PPmin* lowest pulse pressure, *PLR* passive leg raising, *∆SV-PLR* PLR-induced change in stroke volume, *∆PP-PLR* PLR-induced change in radial pulse pressure, *∆VF-PLR* PLR-induced change in the velocity peak of femoral artery flow, *∆PPdim* deep inspiration maneuver-induced change in pulse pressure, *∆VF* respiratory change in velocity peak of femoral artery flow, *∆VFdim* deep inspiration maneuver-induced change in velocity peak of femoral artery flow, *∆PP*_*FB*_ ∆PP during forced inspiratory breathing

**Table 4:** Performance of included studies that addressed echocardiography maneuvers, pulse contour analysis or noninvasive cardiac output monitor (NICOM^®^) to predict fluid responsiveness in spontaneously breathing patients

Author, year	Maneuver	Sens (%)	Spec (%)	PPV (%)	NPV (%)	LR+	LR−	AUC ± SD or (95% CI)
Lamia, 2007 [21]	SVi-PLR ≥ 12.5%	77	100	100	78		0.23	0.95 ± 0.04
Maizel, 2007 [22]	∆CO > 12%	63	89	83	73	6.00	0.40	0.89 ± 0.06
	∆SV > 12%	69	89	85	76	6.00	0.40	0.90 ± 0.06
Biais, 2009 [23]	∆SV-PLR_TE_ > 13%	100	80	91	100	5.00		0.96 ± 0.03
	∆SV-PLR_FloT_ > 16%	85	90	94	75	8.50	0.17	0.92 ± 0.05
Muller, 2012 [24]	cIVC = 40%	70	80	72	83	3.50	0.37	0.77 (0.60–0.88)
	E wave velocity of 0.7	67	90	84	83	6.67	0.37	0.83 (0.68–0.93)
Brun, 2013 [25]	∆VTI-PLR > 12%	75	100	100	79		0.25	0.93 (0.83–1.00)
	VTI ≤ 21 cm	67	100	100	75		0.33	0.82 (0.64–1.00)
Lanspa, 2013 [26]	cIVC ≥ 15%	100	67	62	100	3.00		0.83 (0.58–1.00)
	∆SV ≥ 17%	60	100	100	82		0.40	0.92 (0.73–1.00)
	AoVV ≥ 25%	75	67	50	85	2.25	0.37	0.67 (0.32–1.00)
Airapetian, 2015 [27]	cIVC > 42%	31	97	90	60	9.31	0.71	0.62 (0.66–0.88)
	IVCmax < 2.1 cm	93	33	57	83	1.40	0.21	0.62 (0.49–0.75)
	ΔCO-PLR > 10%	52	87	79	65	3.88	0.56	0.78 (0.66–0.88)
Duus, 2015 [29]	∆SV-PLR	80	61	79	65	2.09	0.31	0.74 (0.65–0.83)
Corl, 2017 [28]	cIVC of 25%	87	81	81	87	4.56	0.16	0.84 (0.77–0.90)

*Sens* sensitivity, *Spec* specificity, *PPV* positive predictive value, *NPV* negative predictive value, *LR* *+* positive likelihood ratio, *LR* *−* negative likelihood ratio, *AUC* area under the receiver operating characteristics curve, *SD* standard deviation, *95% CI* 95% confidence intervals, *PLR* passive leg raising, *SVi-PLR* PLR-induced change in stroke volume index, *ΔCO* change in cardiac output, *∆SV* stroke volume variation, *TE* transthoracic echocardiography, *FloT* FloTrac™, *cIVC* inferior vena cava collapsibility index, *VTI* aortic velocity–time integral, *∆VTI-PLR* VTI variations during PLR, *AoVV* aortic velocity variation, *IVCmax* inferior vena cava maximum diameter, *ΔCO-PLR* change in cardiac output between baseline and after PLR

∆PPV of 52% (AUC ± SD: 0.98 ± 0.03), [16] ∆SV-PLR > 13% (AUC ± SD: 0.96 ± 0.03), [23] ∆PPdim ≥ 12% (AUC ± SD: 0.95 ± 0.05), [19] ∆VFdim ≥ 12% (AUC ± SD: 0.95 ± 0.05) [19] and ∆SV-PLR ≥ 10% (AUC ± SD: 0.94 ± 0.04) [18] showed the highest accuracy to predict fluid responsiveness in spontaneously breathing patients (Fig. 2) (Tables 3 and 4). AoVV ≥ 25% [AUC (95% CI): 0.67 (0.32–1.00)], [26] cIVC > 42% [AUC (95% CI): 0.62 (0.66–0.88)], [27] IVCmax at baseline < 2.1 cm [AUC (95% CI): 0.07 (0.49–0.75)] [27] and ∆SV ≥ 10% [AUC (95% CI): 0.57(0.34-0.78) [17] showed the worst values of accuracy to predict fluid responsiveness (Fig. 2) (Tables 3 and 4).

#### Spontaneous breathing patients without ventilatory support

∆VSP of 52% [AUC ± SD: 0.98 ± 0.03] [16] had the highest accuracy and cIVC > 42% [AUC (95% CI): 0.62 (0.66–0.88)] and IVCmax < 2.1 cm [AUC (95% CI) 0.62 (0.49–0.75)] the worst accuracy to predict fluid responsiveness in spontaneous breathing patients without ventilatory support (12 studies totaling 572 patients) (Additional file 1: Figure S1).

#### Spontaneous breathing with ventilatory support

∆SV-PLR_TE_ > 13% [AUC ± SD: 0.96 ± 0.03] had the highest accuracy, while ∆SV ≥ 10% [AUC (95% CI) 0.57(0.34–0.78)] had the worst accuracy to predict fluid responsiveness in mechanically ventilated patients in a spontaneous mode (3 studies totaling 77 patients) (Additional file 1: Figure S2).

## Discussion

The main finding of this systematic review is that, regardless of intrinsic limitations of each reported maneuver, fluid responsiveness can be assessed in spontaneously breathing patients with acceptable accuracy. Approximately two-thirds (19/29) of reported maneuvers were deemed adequate or excellent to predict fluid responsiveness in spontaneous breathing patients without ventilatory support and 60% (3/5) were deemed excellent in mechanically ventilated patients in a spontaneous mode. Moreover, approximately half of the patients included in this study were not fluid responsive. This finding reinforces the importance of assessing fluid responsiveness in critically ill patients prior to intravascular volume expansion, thus avoiding unnecessary exposure to additional fluids.

In patients with an invasive arterial line in place, dynamic parameters such as ∆PP in association with a maneuver that magnifies cyclic changes in intrathoracic pressures, i.e., deep inspiration or forced inspiratory breathing, represent important tools to assess fluid responsiveness continuously and with minimal inter-rater variability. [19, 20] Echocardiographic maneuvers such as ∆VF, ∆SV, cIVC represent important tools to assess fluid responsiveness in patients without availability of an invasive arterial line [19, 21, 23, 28]. Although it is operator-dependent, echocardiographic is a noninvasive technique that enables fluid responsiveness assessment with good accuracy in spontaneously breathing patients [19, 21, 23, 28]. The main disadvantages of echocardiographic measurements are non-continuous monitoring and high inter-rater variability [18, 24, 27].

Reversible and noninvasive maneuvers that magnify cyclic changes in intrathoracic pressures and on transpulmonary pressure, such as Valsalva or deep inspiration maneuver, in association with ∆PP or echocardiographic measurements, improve the accuracy of the maneuvers without adverse effects, allowing clinicians at the bedside to assess preload dependency [16, 19]. Nevertheless, it is important to emphasize that all reported methods to assess fluid responsiveness in spontaneously breathing patients have limitations [13, 14]. The need of patients cooperation, inability to sustain deep inspiration, presence of pain, intra-abdominal hypertension, major abdominal surgery, low diaphragm strength, higher respiratory rate, low reproducibility and lack of external validation are frequently reported limitations of available methods [16].

Furthermore, transforming a continuous diagnostic index, such as ∆PP and ∆SV, into binary variables (i.e., responders or non-responders) represents an important limitation of all methods to assess fluid responsiveness [37]. The decision of whether to support or avoid volume expansion in patients with intermediate values of continuous diagnostic index could be imprecise (gray zone) [37]. These patients may benefit from a reversible maneuver, such as PLR prior volume expansion to avoid unnecessary exposure to fluids [37].

Our study has limitations. First, it is important to emphasize that the results of this systematic review should be interpreted in the context of the included studies. Furthermore, studies with small sample size, carried out in different clinical scenarios and with a heterogeneous methodology, were included in this systematic review. Finally, systematic reviews are subject to publication bias, which may exaggerate the conclusion of the study if publication is related to the strengths of the results.

## Conclusion

In conclusion, our systematic review suggests that regardless of the limitations of each maneuver, fluid responsiveness could be assessed in spontaneously breathing patients. Further research with adequate sample size and power are necessary to confirm the real accuracy of the different methods available to assess fluid responsiveness in this population of critically ill patients.

## Additional file

**Additional file 1.** A pdf file containing quality of each study was evaluated by the Quality Assessment of Diagnostic Accuracy Studies tool (QUADAS), a receiver operating characteristic curve of methods to assess fluid responsiveness in spontaneous breathing patients without any ventilatory support and in mechanically ventilated patients during a spontaneous mode.

## Abbreviations

95% CI: 95% confidence interval
ΔCO: change in cardiac output
ΔCO-PLR: change in cardiac output between baseline and after PLR
∆PP: pulse pressure variation
∆PPdim: deep inspiration maneuver-induced change in pulse pressure
∆PP-PLR: PLR-induced change in radial pulse pressure
∆PPf: ∆PP during forced inspiratory effort
∆PP_FB_: ∆PP during forced inspiratory breathing
∆PPV: ∆PP during the Valsalva maneuver
∆SP: systolic pressure variation
∆SPf: ∆SP during forced inspiratory effort
∆VSP: ∆SP during the Valsalva maneuver
∆SV: stroke volume variation
∆SV-PLR: passive leg raising-induced change in stroke volume
∆VF: respiratory change in velocity peak of femoral artery flow
∆VFdim: deep inspiration maneuver-induced change in velocity peak of femoral artery flow
∆VF-PLR: PLR-induced change in the velocity peak of femoral artery flow
∆VTI-PLR: VTI variations during PLR
AoVV: aortic velocity variation
AUC: area under the receiver operating characteristics curve
CI: cardiac index
cIVC: inferior vena cava collapsibility index
CO: cardiac output
CVP: central venous pressure
ED: emergency department
HES: hydroxyethyl starch
I.V.: intravenous
ICU: intensive care unit
ITBVI: intrathoracic blood volume index
IVCmax: inferior vena cava maximum diameter
IVCmin: vena cava minimum diameters
LR −: negative likelihood ratio
LR +: positive likelihood ratio
NICOM^®^: noninvasive cardiac output monitor
NPV: negative predictive value
PEEP: positive end-expiratory pressure
PLR: passive leg raising
PPmax: pulse pressure maximal
PPmin: lowest pulse pressure
PPV: positive predictive value
ROC curve: receiver operating characteristics curve
SD: standard deviation
Sens: sensitivity
Spec: specificity
SV: stoke volume
SVi: stroke volume index
SVi-PLR: PLR-induced change in stroke volume index
VTI: aortic velocity–time integral
