# Supplementary material for: Assessment of fluid responsiveness in spontaneously breathing patients: a systematic review of literature
Source: Ann Intensive Care. 2018 Feb 9;8:21. doi: 10.1186/s13613-018-0365-y (PMC5807252; doi:10.1186/s13613-018-0365-y)
Supplement: Supplementary file 1 — Additional file 1. A pdf file containing quality of each study was evaluated by the Quality Assessment of Diagnostic Accuracy Studies tool (QUADAS), a receiver operating characteristic curve of methods to assess fluid responsiveness in spontaneous breathing patients without any ventilatory support and in mechanically ventilated patients during a spontaneous mode. [file 13613_2018_365_MOESM1_ESM.docx]

**ASSESSMENT OF FLUID RESPONSIVENESS IN SPONTANEOUSLY BREATHING PATIENTS: A SYSTEMATIC REVIEW OF LITERATURE**

**Additional file 1**

Authors: Renato Carneiro de Freitas Chaves, Thiago Domingos Corrêa, Ary Serpa Neto, Bruno de Arruda Bravim, Ricardo Luiz Cordioli, Fabio Tanzillo Moreira, Karina Tavares Timenetsky and Murillo Santucci Cesar de Assunção.

**RESULTS**

Quality of the studies analyzed

| The QUADAS tool | | | | | | | | | | | | | | |  |
| --- | --- | --- | --- | --- | --- | --- | --- | --- | --- | --- | --- | --- | --- | --- | --- |
| Studies | **1** | **2** | **3** | **4** | **5** | **6** | **7** | **8** | **9** | **10** | **11** | **12** | **13** | **14** | |
| Corl, 2017 | Y | Y | Y | Y | Y | Y | Y | Y | Y | U | U | Y | Y | Y | |
| Airapetian, 2015 | Y | Y | Y | Y | Y | Y | N | Y | Y | U | U | Y | Y | Y | |
| Duus, 2015 | Y | Y | Y | U | Y | Y | N | Y | Y | U | U | Y | Y | Y | |
| Hong, 2014 | Y | Y | Y | Y | Y | Y | Y | Y | Y | U | U | Y | Y | Y | |
| Lanspa, 2013 | Y | Y | Y | Y | Y | Y | N | Y | Y | U | U | Y | Y | Y | |
| Brun, 2013 | Y | Y | Y | Y | Y | Y | N | Y | Y | U | N | Y | Y | Y | |
| Muller, 2012 | Y | Y | Y | Y | Y | Y | Y | Y | Y | U | U | Y | Y | Y | |
| Préau, 2012 | Y | Y | Y | Y | Y | Y | Y | Y | Y | U | U | Y | Y | Y | |
| Préau, 2010 | Y | Y | Y | Y | Y | Y | N | Y | Y | U | U | Y | Y | Y | |
| Monnet, 2009 | Y | Y | Y | Y | Y | Y | Y | Y | Y | U | U | Y | Y | Y | |
| Monge García, 2009 | Y | Y | Y | Y | Y | Y | Y | Y | Y | U | U | Y | Y | Y | |
| Biais, 2009 | Y | Y | Y | Y | Y | Y | N | Y | Y | U | U | Y | Y | Y | |
| Soubrier, 2007 | Y | Y | Y | Y | Y | Y | Y | Y | Y | U | U | Y | Y | Y | |
| Maizel, 2007 | Y | Y | Y | Y | Y | Y | N | Y | Y | U | U | Y | Y | Y | |
| Lamia, 2007 | Y | Y | Y | Y | Y | Y | N | Y | Y | U | U | Y | Y | Y | |

Y: Yes; N: No; U: Unclear

1. Was the spectrum of patients representative of the patients who will receive the test in practice?

2. Were selection criteria clearly described?

3. Is the reference standard likely to correctly classify the target condition?

4. Is the time period between reference standard and index test short enough to be reasonably sure that the target condition did not change between the two tests?

5. Did the whole sample or a random selection of the sample, receive verification using a reference standard of diagnosis?

6. Did patients receive the same reference standard regardless of the index test result?

7. Was the reference standard independent of the index test (i.e. the index test did not form part of the reference standard)?

8. Was the execution of the index test described in sufficient detail to permit replication of the test?

9. Was the execution of the reference standard described in sufficient detail to permit its replication?

10. Were the index test results interpreted without knowledge of the results of the reference standard?

11. Were the reference standard results interpreted without knowledge of the results of the index test?

12. Were the same clinical data available when test results were interpreted as would be available when the test is used in practice?

13. Were uninterpretable/ intermediate test results reported?

14. Were withdrawals from the study explained?

**Figure S1**. Receiver operating characteristics curve of methods to assess volume responsiveness in spontaneous breathing patients without any ventilatory support.


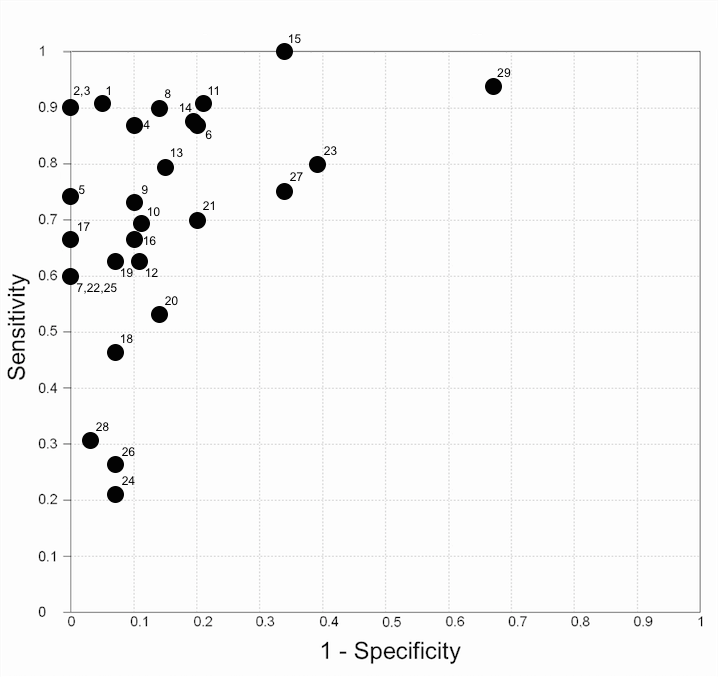


**Legend figure S1**. 1 = ∆PPV of 52%; 2 = ∆PPdim ≥12%; 3 = ∆VFdim ≥12%; 4 =∆SV-PLR ≥10%; 5 = ∆VTI-PLR >12%; 6 = ∆VF-PLR ≥8%; 7 = ∆SV ≥17%; 8 = ∆PP_FB_  =13.7%; 9 = ∆VSP of 30%; 10 = ∆SV >12%; 11 = PPmin of 45mmHg; 12 = ∆CO >12%; 13 = ∆PP-PLR ≥9%; 14 = cIVC of 25%; 15 = cIVC ≥15%; 16 = E wave velocity of 0.7; 17 = VTI ≤21cm; 18 = ∆SP of 9%; 19 = ∆PP of 12%; 20 = ΔCO-PLR >10%; 21 = cIVC =40%; 22 = ∆VF ≥10%; 23 = ∆SV-PLR; 24 = ∆PPf of 33%; 25 = ∆PP ≥10%; 26 = ∆SPf of 30%; 27 = AoVV ≥25%; 28 = cIVC >42%, 29 = IVCmax <2.1cm.

**Figure S2**. Receiver operating characteristics curve of methods to assess volume responsiveness in mechanical ventilation patients during spontaneous mode


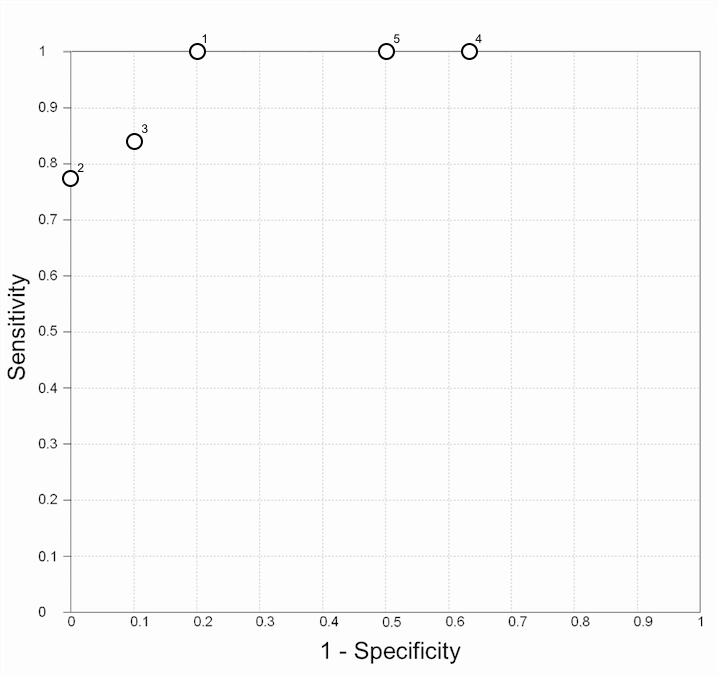


**Legend figure S2**. 1 = ∆SV-PLR_TTE_ >13%; 2 = SVi-PLR ≥12.5%; 3 = ∆SV-PLR_FloT_ >16%; 4 = ∆PP ≥11%; 5 = ∆SV≥10%.
